# Single-channel attention classification algorithm based on robust Kalman filtering and norm-constrained ELM

**DOI:** 10.3389/fnhum.2024.1481493

**Published:** 2025-01-09

**Authors:** Jing He, Zijun Huang, Yunde Li, Jiangfeng Shi, Yehang Chen, Chengliang Jiang, Jin Feng

**Affiliations:** ^1^School of Management, Guilin University of Aerospace Technology, Guilin, China; ^2^School of Electronic Engineering and Automation, Guilin University of Electronic Technology, Guilin, China; ^3^Biomedical and Artificial Intelligence Laboratory, Guilin University of Aerospace Technology, Guilin, China; ^4^School of Automation Science and Engineering, South China University of Technology, Guangzhou, China; ^5^Student Affairs Office, Guilin Normal College, Guilin, China

**Keywords:** brain-computer interfaces, attentional state, robust Kalman, convex optimization, norm-ELM

## Abstract

**Introduction:**

Attention classification based on EEG signals is crucial for brain-computer interface (BCI) applications. However, noise interference and real-time signal fluctuations hinder accuracy, especially in portable single-channel devices. This study proposes a robust Kalman filtering method combined with a norm-constrained extreme learning machine (ELM) to address these challenges.

**Methods:**

The proposed method integrates Discrete Wavelet Transformation (DWT) and Independent Component Analysis (ICA) for noise removal, followed by a robust Kalman filter enhanced with convex optimization to preserve critical EEG components. The norm-constrained ELM employs L1/L2 regularization to improve generalization and classification performance. Experimental data were collected using a Schulte Grid paradigm and TGAM sensors, along with publicly available datasets for validation.

**Results:**

The robust Kalman filter demonstrated superior denoising performance, achieving an average AUC of 0.8167 and a maximum AUC of 0.8678 on self-collected datasets, and an average AUC of 0.8344 with a maximum of 0.8950 on public datasets. The method outperformed traditional Kalman filtering, LMS adaptive filtering, and TGAM’s eSense algorithm in both noise reduction and attention classification accuracy.

**Discussion:**

The study highlights the effectiveness of combining advanced signal processing and machine learning techniques to improve the robustness and generalization of EEG-based attention classification. Limitations include the small sample size and limited demographic diversity, suggesting future research should expand participant groups and explore broader applications, such as mental health monitoring and neurofeedback.

## Introduction

Attention, characterized by distinct EEG activity patterns, refers to the ability to focus on specific stimuli (Wang et al., 2021). In everyday life, differences in attention levels affect performance in many areas. With advancements in neuroscience, EEG signals are increasingly utilized for analyzing attention states. For instance, Al-Nafjan and Aldayel (2022) applied EEG-based attention assessments in online learning environments, identifying changes in student attentiveness. In the music domain, EEG signals detect attentiveness to different music, facilitating personalized recommendations (Zhang et al., 2021). In the driving field, attention signals assess driving fatigue. Kashihara (2017) classified brain activity from wavelet signals during attention tasks to identify road signs, developing a system to prevent car accidents. Similarly, Atilla and Alimardani (2021) monitored drivers’ attention to detect fatigue. Du et al. (2017) applied physiological signals such as EEG and EOG to driving fatigue detection and proposed a multimodal method combining partial EEG and frontal EEG to enhance driving fatigue detection. In healthcare, EEG-based assessments effectively diagnose and treat attention deficit hyperactivity disorder (ADHD) in children (Miranda et al., 2020). Brain-Computer Interface (BCI) technology enables direct interaction with external devices using electroencephalogram (EEG) signals (Thomas, 2023).

This study focuses on the use of single-channel EEG for attention classification, where the quality of the signal is a key factor in achieving accurate results. Compared to multi-channel EEG systems, single-channel approaches offer a more convenient and faster solution, beneficial for expanding BCI applications.

### Related work

Studies have shown that EEG signals vary under different attention tasks. Wang et al. (2021) extracted significant patterns from EEG signals and proposed an attention-based multi-scale convolutional neural network-dynamic graph convolutional network (AMCNN-DGCN) model, tested in fatigue driving environments. Changes in attention states are related to fluctuations in specific EEG frequency bands (Kamiński et al., 2012; Klimesch et al., 2012; Arns et al., 2013; Liu et al., 2013; Pitchford and Arnell, 2019). Increased Beta (*β*) wave activity indicates heightened attention. High-frequency bands in Delta (*δ*), Theta (*θ*), Alpha (*α*), and Beta (β) rhythms are commonly used as EEG features. Huang et al. (2016) used Granger causality bias coherence analysis for different psychological tasks, such as gaming and resting, to measure local and global network efficiency. They found that local efficiency in the β band was higher during tasks and lower during rest, while global efficiency showed the opposite trend. This distinction can differentiate attention tasks. Common EEG classification features include frequency band energy and power spectral density (Tang and Huang, 2020). These applications demonstrate the potential of BCI technology in studying and monitoring attention.

However, existing research faces challenges such as noise interference common in EEG signals, which affects the accuracy of attention detection (Jiang et al., 2019). Traditional methods for removing EEG artifacts, such as wavelet with higher-order statistics (Castellanos and Makarov, 2006), independent component analysis (ICA) (Jung et al., 2001), and principal component analysis (PCA) (Lagerlund et al., 1997), require multiple EEG channels or simultaneous electrooculography (EOG) signal acquisition, which is impractical for portable single-channel EEG devices. Recent advancements have addressed these limitations. Chintala and Thangaraj (2020) recorded horizontal and vertical electrooculogram (EOG) signals as reference signals and employed finite impulse response filters for processing. Somers et al. (2018) proposed an algorithm based on multichannel Wiener filtering, which substituted the pseudo-signal covariance matrix with a low-rank approximation based on generalized eigenvalue decomposition. However, when using filtering methods, the EEG is contaminated by eye signals, and the EOG reference signal is also contaminated by the EEG, leading to a bidirectional contamination issue. To overcome these drawbacks, Ge et al. (2018) proposed a method using higher-order statistical tensors through an Underdetermined Blind Source Separation (UBSS) model to separate artifacts from EEG signals. Saini et al. (2020) proposed a framework based on Variational Mode Decomposition (VMD) and turning-point counting, validated across three standard databases. Research indicates that most eye artifact removal techniques require the collection of multiple EEG channels or simultaneous collection of EOG signals as references, which is not suitable for portable single-channel EEG devices. Despite their effectiveness, these methods often lack real-time processing capabilities, leading to delays in practical applications. Traditional adaptive filtering methods, such as the Least Mean Squares (LMS) algorithm (Mowla et al., 2018) and Kalman filter (Hesar and Mohebbi, 2021), face challenges with real-time, nonlinear, and non-smooth signals. The Kalman filter’s adaptive adjustment capabilities optimize filter parameters based on real-time data, making it suitable for dynamic EEG signals, but it may misclassify sparse noise as Gaussian noise.

This study proposes an innovative and robust method for accurately estimating attention levels in EEG signals, focusing on real-time noise reduction and signal stability. To address real-time noise reduction and signal fluctuations, we propose a novel approach combining a robust Kalman filter for noise removal with a norm-constrained Extreme Learning Machine (ELM) for handling dynamic attention changes. The robust Kalman algorithm preserves critical EEG components through signal preprocessing and optimized estimation. The norm-constrained ELM introduces nonlinear mapping for correct estimation, retaining relevant dynamic information and improving attention assessment accuracy and stability.

## Methods

### Data acquisition

**Figure 1:**
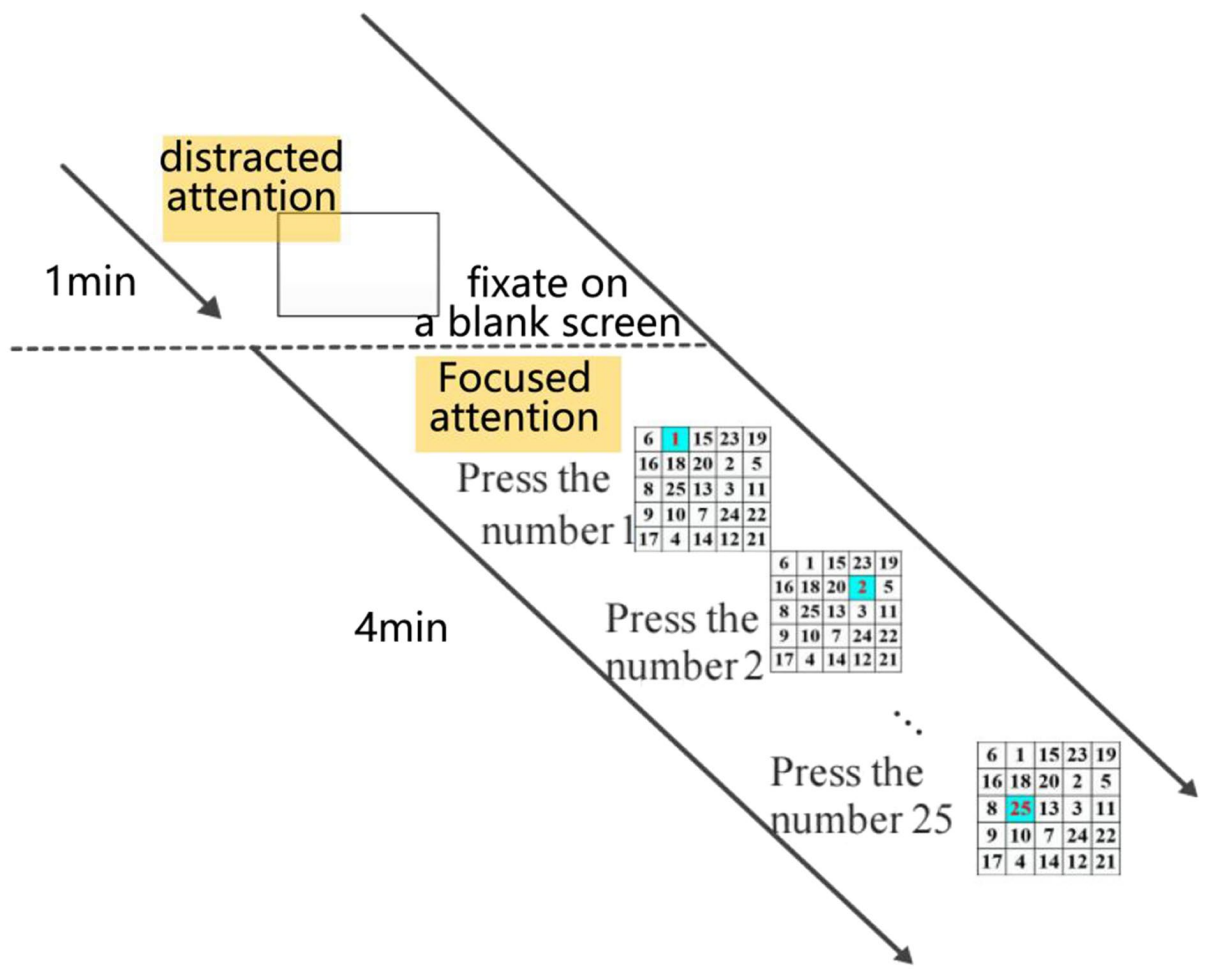
Experimental procedure for data collection.

Since attention signals are spontaneous EEG, a specific experimental paradigm is required to elicit EEG signals based on attention mechanisms. In this study, eight subjects participated in the experiment. Data collection was conducted using a 5 × 5 Schulte Grid, as shown in Figure 1.

The Schulte Grid is a classic psychological experimental tool commonly used to measure individual attention, concentration, and visual perception speed. By asking participants to find randomly arranged numbers in turn, the Schulte Grid tests participants’ attention concentration, visual field range, and visual search ability. It is widely used in the field of attention assessment and is especially suitable for assessing individual attention fluctuations in a short period of time (Lu et al., 2022). Schulte Grids are based on a visual search task, which has been demonstrated by multiple studies to reliably induce attention-related brain wave changes, particularly attention states associated with alpha and beta waves. It is simple to operate and easy to implement, making it suitable as an experimental paradigm for EEG signal acquisition. Therefore, future research could consider using more complex experimental paradigms, such as the Continuous Performance Test (CPT) or the N-back task, which can more systematically induce sustained attention and test the stability of attention under increased cognitive load.

The experimental paradigm is as follows: To maintain focus, subjects were allowed to rest before the test began. During the test, subjects sequentially identified the positions of Arabic numerals from 1 to 25 on the Schulte Grid. The experiment included a 4-min stimulation period followed by a 1-min interval. Each subject completed three phases, totaling 15 min.

The TGAM sensor was used with three contact points: EEG (EEG acquisition point), REF (reference point), and GND (ground point). The TGAM default port rate is 57,600, with approximately 513 packets per second, and raw EEG data is output at 512 Hz (Wu and Xie, 2020). The data collection process was conducted as follows: A personal computer (PC) served as the data processing center, receiving packets from the EEG module TGAM via a Bluetooth serial communication protocol. The PC paired with the TGAM biosensing chip module via Bluetooth to facilitate data transmission.

This paper also utilized experimental data for monitoring individual attention published by Acı et al. (2019). Each file contained data obtained from the EEG device during the experiment, with raw data sampled at 128 Hz. This study used only the AF3 data channel from 0–10 min (focused) and 10–20 min (unfocused).

### Algorithm overview

**Figure 2:**
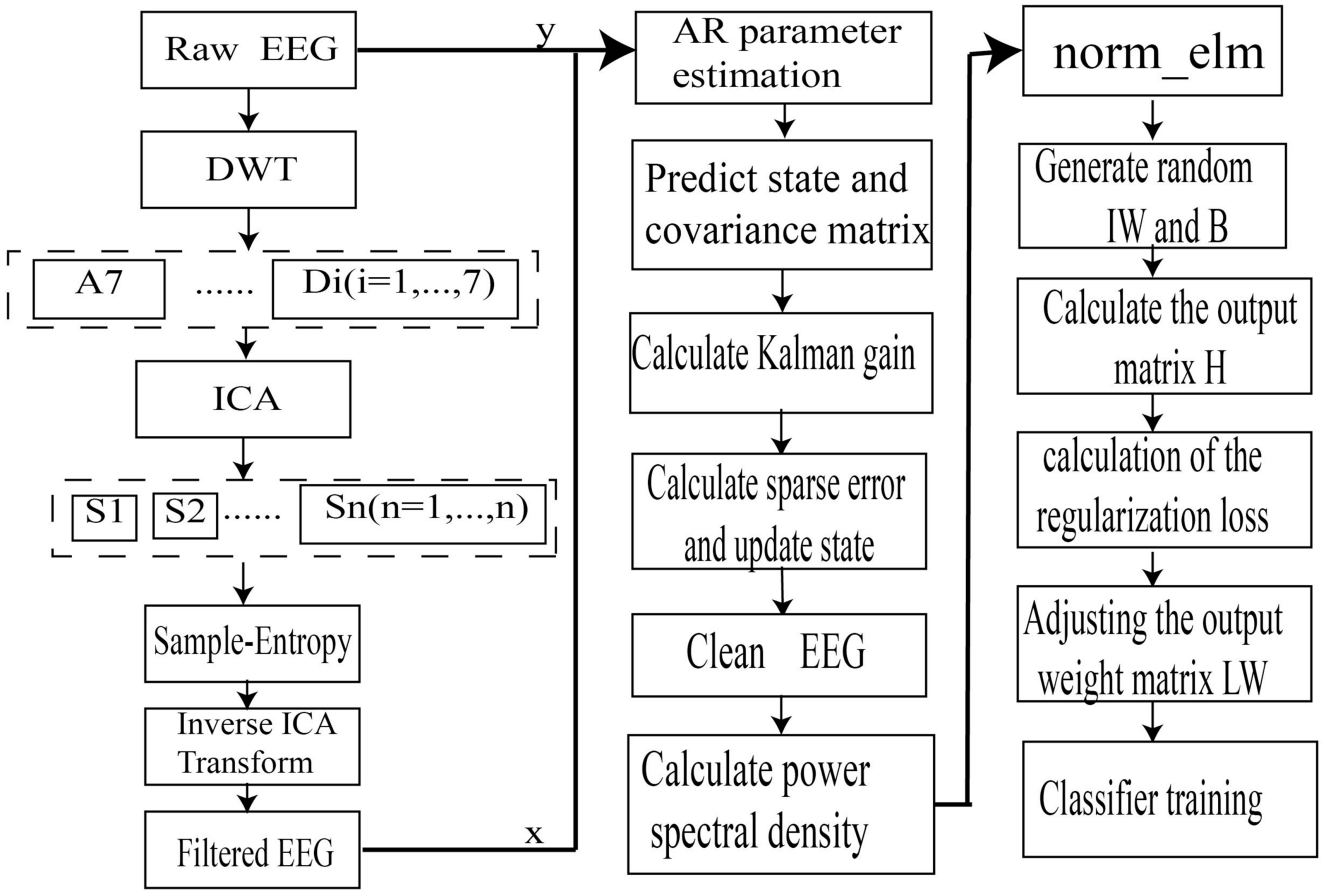
System flowchart.

Conventional EEG processing algorithms often overlook the dynamic interdependencies within EEG signal changes, leading to inaccurate recognition of attention states. Therefore, our study introduces a robust Kalman filter method for dynamic attention detection. The Kalman filter’s ability to update signal estimates in real-time allows for the dynamic capture of changes in attentional mechanisms, addressing the shortcomings of traditional methods in terms of real-time performance and adaptability. The algorithm designed in our study is depicted in Figure 2.

The proposed algorithm includes three main improvements. First, the preprocessing stage combines Discrete Wavelet Transformation (DWT) and Independent Component Analysis (ICA) to better remove noise and retain dynamic useful information from EEG signals. Second, an improved Kalman filter algorithm integrates convex optimization techniques and Bayesian theory to enhance signal estimation, utilizing an augmented reality model and incorporating sparse noise control. Third, L1/L2 norm regularization in the Extreme Learning Machine (ELM) algorithm is chosen for its benefits in simplifying the model and improving generalization capability. Specifically, L1 regularization aids in feature selection, while L2 regularization helps in learning more effective features. By incorporating these regularization constraints, the ELM algorithm’s generalization performance is significantly enhanced.

### Denoising

This study employs the Independent Component Analysis (ICA) algorithm to decompose and evaluate EEG signals. The Sample Entropy algorithm (Liu et al., 2023) calculates the entropy of the decomposed signal components to determine the entropy information of each component. Removing lower-entropy components effectively eliminates noise and artifacts.

### Robust Kalman target tracking model

In Equation 1, the system was first expressed as a convex optimization problem by constructing an attention state tracking model for single-channel EEG, introducing tuning parameters to balance data fit and noise sparsity. The optimal estimate is obtained by optimizing the objective function, dynamically updating the state estimate of the EEG by considering both prior and measurement information to improve the system’s adaptive ability. Specifically, the attention level tracking model is expressed as:


(1) $$\begin{array}{l} x_{t}=Ax_{t-1}+w_{t}\\ y_{t}=Cx_{t}+v_{t}+z_{t} \end{array}$$

where $x_{t}\in R^{N}$ denotes the clean EEG at time *t*, $y_{t}\in R^{M}$ is the original EEG signals at time *t*, *A* is the dynamic change matrix of the brain’s attentional state, *C* maps state vectors to observation vectors, $w_{t}\in R^{N}$ denotes the noisy EEG, $v_{t}\in R^{M}$ represents measurement noise, and $z_{t}\in R^{M}$ denotes sparse noise, which can cause signal distortion, introduce artifacts, and reduce the signal-to-noise ratio.

The estimation of attention level involves predicting the next time point’s attention level based on known original EEG signals $x_{t+1}$ . To obtain the optimal probability estimate of $p\left(x_{t+1}|y_{t}\right)$ , the maximum likelihood estimation theory (Sur and Candès, 2019) constructs the likelihood function $L\left(y_{t}|x_{t+1}\right)$ :


(2) $$\begin{array}{l} L\left(y_{t}|x_{t+1}\right)=\frac{p\left(x_{t+1}\right)p\left(v_{t}\right)}{p\left(x_{t+1}\right)}\\ =\frac{1}{{\left(\sqrt{2\pi }\right)}^{m}|V{|}^{1/2}}\exp\left(-\frac{1}{2}{v_{t}}^{T}V^{-1}v_{t}\right) \end{array}$$

where *V* denotes the covariance matrix of the measurement noise $v_{t}$ , $V^{-1}$ reflecting the statistical properties of the observation noise. According to the full probability formula, the maximum likelihood function $L\left(x_{t+1}\right)$ is expressed as:


(3) $$\begin{array}{l} L\left(x_{t+1}\right)=\frac{1}{{\left(\sqrt{2\pi }\right)}^{n}|V{|}^{1/2}}\\ \exp\left({\left(x-{\hat{x}}_{t|t-1}\right)}^{T}V^{-1}\left(x-{\hat{x}}_{t|t-1}\right)\right) \end{array}$$

A tuning parameter λ is introduced to balance data fitting and noise sparsity $z_{t}$ . The conditional probability $p\left(x_{t+1}|y_{t}\right)$ is obtained by minimizing the power exponent in Equations 2, 3 to calculate the optimal estimate:


(4) $$\begin{array}{l} \min_{x_{t}v_{t}y_{t}}{v_{t}}^{T}V^{-1}v_{t}+{\left(x-{\hat{x}}_{t|t-1}\right)}^{T}\overset{-1}{\sum }\left(x-{\hat{x}}_{t|t-1}\right)+\lambda ||z_{t}|{|}^{1}\\ s.t.\;y_{t}=Cx+v_{t}+z_{t} \end{array}$$

In Equation 4, from $v_{t}=y_{t}-Cx-z_{t}$ , define the optimization objective function $F\left(x,z_{t}\right)$ as:


(5) $$\begin{array}{l} F\left(x,z_{t}\right)={\left(y_{t}-Cx-z_{t}\right)}^{T}V^{-1}\left(y_{t}-Cx-z_{t}\right)+{\left(x-{\hat{x}}_{t|t-1}\right)}^{T}V^{-1}\\ \left(x-{\hat{x}}_{t|t-1}\right)+\lambda ||z_{t}|{|}^{1} \end{array}$$

Applying the partial derivative to x in Equation 5, one obtains.


(6) $$\begin{array}{l} \frac{\partial F\left(x,z_{t}\right)}{\partial x}=2C^{T}V^{-1}Cx-2C^{T}V^{-1}y_{t}+2C^{T}V^{-1}z_{t}\\ +2\overset{-1}{\sum }x-2\overset{-1}{\sum }{\hat{x}}_{t} \end{array}$$

The optimal state estimate is obtained by setting Equation 6 to zero:


(7) $$x={\hat{x}}_{t}+\sum C^{T}{\left(V+C\sum C^{T}\right)}^{-1}\;\left(e_{t}-z_{t}\right)$$

In Equation 7, $e_{T}=Z_{T}-c{\hat{x}}_{t}$ , *c* is a constant; by updating the real-time estimate of non-stationary noise at each iteration, the optimal estimate $\hat{x}$ of attention level is obtained by weighting the observed and predicted values. The improved convex optimization Kalman expression is:


(8) $$\begin{array}{l} \min\left(e_{t}-z_{t}\right){\left(I-C\sum C^{T}{\left(V+C\sum C^{T}\right)}^{-1}\right)}^{T}\\ V^{-1}\left(I-C\sum C^{T}{\left(V+C\sum C^{T}\right)}^{-1}\right)\\ +\sum C^{T}{\left(V+C\sum C^{T}\right)}^{-1T}\sum^{-1}\sum C^{T}\\ {\left(V+C\sum C^{T}\right)}^{-1}\left(e_{t}-z_{t}\right)+\lambda ||z_{t}|{|}^{1} \end{array}$$

In Equation 8, the sparse noise $z_{t}$ is the only variable. Controlling $z_{t}$ aiding in the detection and analysis of attention-related signal features while reducing noise effects.

### Norm-constrained ELM classifier

In the second step, the ELM algorithm with L1/L2 norm constraints is used to correct the EEG estimates. The EEG state estimates serve as inputs to construct an ELM model with T hidden layer nodes, introducing regularization constraints to improve generalization. The specific steps are:


(9) $$\begin{array}{l} \Phi \left(w_{1},w_{2},\ldots ,w_{T},b_{1},b_{2},\ldots ,b_{T},a_{1},a_{2},\ldots ,a_{T}\right)\beta =\\ {\left[\begin{array}{ccc} g\left(w_{1}{a_{1}}^{T}+b_{1}\right) & \ldots & g\left(w_{T}{a_{1}}^{T}+b_{T}\right)\\ \vdots & \ddots & \vdots \\ g\left(w_{1}{a_{N}}^{T}+b_{1}\right) & \ldots & g\left(w_{T}{a_{N}}^{T}+b_{T}\right) \end{array}\right]}_{N\times T}*\beta =t\\ \beta ={\left[\beta_{1},\beta_{2},\ldots ,\beta_{T}\right]}^{T},t={\left[t_{1},t_{2},\ldots ,t_{N}\right]}^{T} \end{array}$$

In Equation 9, where Φ is the hidden layer output matrix, β is the hidden layer weight matrix, and *t* is the training set target matrix. The output weights connecting the hidden and output layers $\beta_{j}$ are solved by minimizing the squared difference algorithm with the objective function (Yıldırım and Özkale, 2023):


(10) $$\hat{\beta }=\arg\min\frac{1}{2}||\Phi \beta -t|{|}_{2}^{2}=\Phi^{†}t$$

In Equation 10, where: $\Phi^{†}$ is the pseudo-inverse; $\hat{\beta }$ is the estimated value of the target output;


(11) $$\hat{\beta }=\arg\min\frac{1}{2}||\Phi \beta -t|{|}_{2}^{2}+\lambda_{1}||\beta |{|}^{1}+\lambda_{2}||\beta |{|}^{2}$$

The values of $\lambda_{1}$ and $\lambda_{2}$ , which are regularization hyperparameters constrained by L1 and L2 norms, are primarily used to enhance the generalization capability and stability of the model. Formula 11 is defined in the following matrix form:


(12) $$\Phi^{*}=\sqrt{1+\lambda_{2}}\left(\begin{array}{c} \Phi \\ \sqrt{\lambda_{2}}I \end{array}\right),t^{*}=\left(\begin{array}{c} t\\ 0 \end{array}\right)$$

Let $\gamma =\lambda_{1}/\sqrt{\lambda_{1}+\lambda_{2}},\beta^{*}=\sqrt{1+\lambda_{2}}\beta$ , and combined with formula 12, then formula 11 can be expressed as shown below.


(13) $$\begin{array}{l} \hat{\beta }=\frac{1}{\sqrt{1+\lambda_{2}}}{\hat{\beta }}^{*}\\ =\frac{1}{\sqrt{1+\lambda_{2}}}\arg\min\frac{1}{2}||\Phi^{*}\beta^{*}-t^{*}|{|}_{2}^{2}+\gamma ||\beta^{*}|{|}^{1} \end{array}$$

Since $\lambda_{2}$ is a set constant, Equation 17 can be further expanded to get.


(14) $$\begin{array}{l} \hat{\beta }=\frac{1}{2}\Phi^{*}\beta^{*}-t^{*T}\Phi^{*}\beta^{*}-t^{*}+\lambda \beta^{*T}\beta^{*}\\ =\frac{1}{2}\beta^{*T}\Phi^{*T}\Phi^{*}\beta^{*}-\beta^{*T}\Phi^{*T}t^{*}-t^{*T}\Phi^{*}\beta^{*}\\ +t^{*T}t^{*}+\lambda \beta^{*T}\beta^{*} \end{array}$$

In Equation 14, where the $\gamma ,\beta^{*}$ parameter is a constraint function that mainly corrects the estimate of the attentional state.

Equation 13 takes the partial derivative of $\beta^{*}$ and makes the derivative zero, resulting in a weight estimate that minimizes the number of targets as in Equation 15.


(15) $${\hat{\beta }}^{*}={\left(\Phi^{*T}\Phi^{*}+\lambda I\right)}^{-1}\Phi^{*T}t^{*}$$

The final robust Kalman is corrected to obtain an expression for the estimate as in Equation 16:


(16) $$\mathrm{output}={\hat{\beta }}^{*T}\Phi^{*T}$$

To evaluate the proposed algorithm’s performance, we conducted experiments on both self-collected and public EEG datasets, focusing on key evaluation metrics such as AUC (Area Under the Curve) and ROC curve (Receiver Operating Characteristic Curve) to assess classification accuracy (Nahm, 2022). Below, we provide a detailed comparison of the results across different algorithms.

By definition, the AUC can be obtained by summing the area of each part under the ROC curve. The ROC curve is assumed to form $\left(x_{1}=0,x_{m}=1\right)$ by connecting points in sequence with coordinates $\left\{\left(x_{1},y_{1}\right),\left(x_{2},y_{2}\right),\ldots ,\left(x_{m},y_{m}\right)\right\}$ , the AUC is calculated as:


(17) $$AUC=\frac{1}{2}\overset{m-1}{\underset{i=1}{\sum }}\left(x_{i+1}-x_{i}\right)\left(y_{i}+y_{i+1}\right)$$

## Result

### Denoising experiment

**Figure 3:**
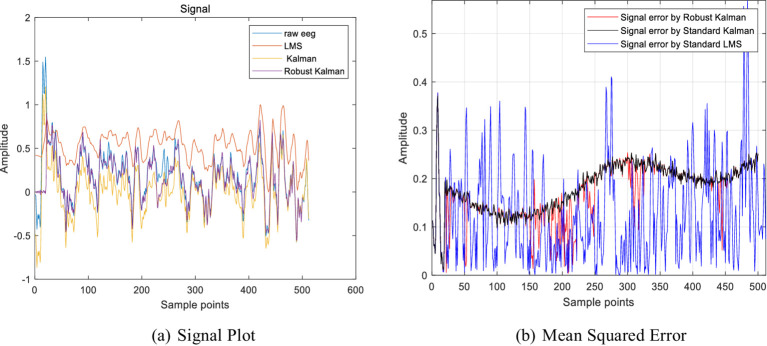
Results of denoising experiments. **(A)** Signal plot. **(B)** Mean squared error.

In the experiment, a small amount of sparse noise was added to the original signal. The denoising capabilities of different algorithms were then compared to validate the effectiveness of the proposed algorithm. The experimental results showed that the robust Kalman model proposed in this study had the highest similarity with the original signal, indicating that it was least affected by sparse noise. The results of the denoising experiment are shown in Figure 3A. By calculating the mean square error (MSE) values (Papaioannou et al., 2021) for various comparison algorithms, it is evident that the proposed algorithm can accurately track the signal and provides superior filtering performance, as shown in the MSE results in Figure 3B. The mean squared error (MSE) values after applying the Kalman filter fluctuate around an average of 0.2. This is because, when sparse noise is present in the signal data, the traditional Kalman algorithm mistakenly identifies other noise signals as Gaussian noise, thereby affecting the denoising accuracy. LMS adaptive filtering exhibited larger MSE fluctuations due to over-filtering. In contrast, the MSE values after using the robust Kalman filter are lower than those of the traditional Kalman filter. This further indicates that the robust Kalman model proposed in this study is capable of removing noise while retaining as much effective information from the original signal as possible.

### Assessing effectiveness

**Figure 4:**
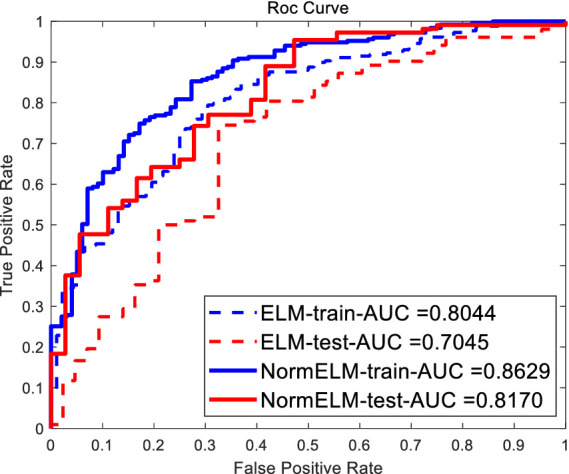
Comparison results of different ELM algorithms.

In the experiment, considering that Extreme Learning Machine (ELM) uses random weights as initial model weights, ELM may overfit these noisy points due to the presence of outliers in some data, thereby lacking an accurate fit to the true data situation. To further prevent overfitting in ELM, which can decrease the model’s generalization performance, regularization terms (L1 or L2) were added to constrain the model parameters, reducing model complexity. Figure 4 compares the experimental results of the conventional ELM and the ELM algorithm based on L1/L2 regularization terms.

**Figure 5:**
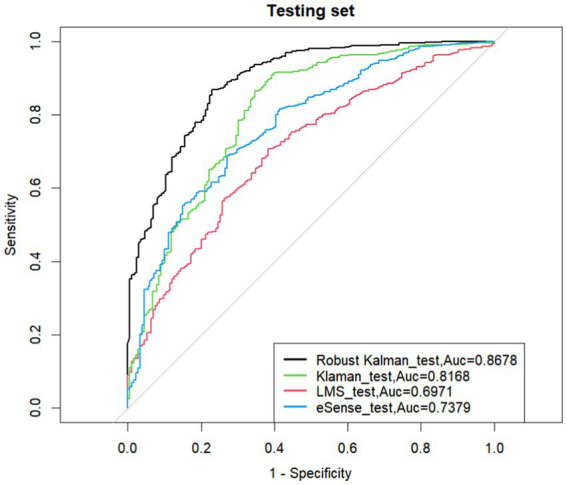
ROC curves of different algorithms.

As shown in the experimental results in Figure 5, the data processed by the robust Kalman algorithm achieved an AUC of 0.8678 after applying the ELM algorithm with L1/L2 regularization. In comparison, the traditional Kalman filter algorithm had an AUC of 0.8168, the traditional adaptive filtering algorithm had an AUC of 0.6971, and the eSense algorithm in the TGAM module with the robust Kalman algorithm had an AUC of 0.7379.

If the ROC curve of one learner is completely “enclosed” by the curve of another learner, the performance of the latter is superior. If the ROC curve of the two learners crosses, it is difficult to claim the superiority of the two; in that case, the reasonable criterion is to compare the area under the ROC curve, AUC.

In this study, EEG signals were collected from eight participants, and different processing methods were applied to each participant’s data. The experimental results are shown in Table 1. The results indicate that the proposed robust Kalman algorithm achieved an average AUC of 0.8167 on the test sets of the 8 participants, with the difference between the training and test set AUCs ranging from 0.02 to 0.04. In contrast, the traditional Kalman filter algorithm achieved an average AUC of 0.7417 on the test sets, the adaptive LMS algorithm achieved an average test set AUC of 0.6214, and the eSense algorithm (López-Ahumada et al., 2023) integrated into the TGAM module achieved an average test set AUC of 0.7011. This further demonstrates that the proposed robust Kalman algorithm has strong classification performance and effectively suppresses overfitting.

**Table 1:** AUC results for different algorithms on 8 subjects.

AUC	Robust Kalman train	Robust Kalman test	Kalman train	Kalman test	LMS train	LMS test	eSense train	eSense test
1	0.8629	0.8170	0.7528	0.7482	0.7054	0.6971	0.7289	0.6913
2	0.8127	0.8055	0.7825	0.7782	0.6606	0.6203	0.7816	0.7454
3	0.8832	0.8658	0.7789	0.7523	0.6100	0.5548	0.8139	0.7772
4	0.7455	0.7265	0.6517	0.6191	0.5336	0.5188	0.7506	0.6640
5	0.8767	0.8514	0.8172	0.7852	0.7204	0.7034	0.7293	0.6715
6	0.8172	0.8058	0.7667	0.7416	0.6236	0.6096	0.7796	0.6774
7	0.9065	0.8678	0.8425	0.8168	0.7241	0.6971	0.7492	0.7379
8	0.8110	0.7937	0.7062	0.6922	0.5891	0.5703	0.6666	0.6442
ave	0.8395	0.8167	0.7623	0.7417	0.6459	0.6214	0.7500	0.7011

Additionally, to further validate the performance of the proposed algorithm on other datasets, this study used a publicly available datasets published by Acı et al. (2019) on Kaggle. This dataset contains data related to two states (focused and unfocused) of individuals. For this study, only the AF3 channel data from the public dataset was used. As shown in Table 2, the robust Kalman algorithm achieved an average AUC of 0.8344 on the test sets of three groups of subjects. This indicates that the proposed algorithm also performs well on other datasets.

**Table 2:** AUC results of different algorithms on public data sets.

AUC	Robust Kalman train	Robust Kalman test	Kalman train	Kalman test	LMS train	LMS test
1	0.8143	0.7777	0.7620	0.7460	0.6537	0.6283
2	0.9196	0.8950	07155	0.6791	0.6768	0.5845
3	0.8565	0.8305	0.6581	0.6389	0.6270	0.6136
ave	0.8635	0.8344	0.85473	0.6880	0.6525	0.6088

The experimental results show that the robust Kalman algorithm produces an average AUC of 0.8167 and a maximum AUC of 0.8678 on the in-house collected datasets. It also showed good performance on the public datasets, with an average AUC of 0.8344 and a maximum AUC of 0.8950. These results highlight the effectiveness of the algorithm in accurately estimating levels of attention, significantly surpassing conventional methods such as LMS adaptive filtering and the conventional Kalman filter. The study demonstrates that the robust Kalman algorithm proposed significantly improves the accuracy of attention level estimation, showing superior classification accuracy and robust model generalization capability. These results offer strong evidence for the practical implementation of this novel approach in tasks related to monitoring attention states.

## Discussion

This study addresses a critical gap in the current field of attention tracking, specifically in evaluating and monitoring attention levels using electroencephalogram (EEG) signals. We present an effective and practical method for estimating attention levels from EEG signals. Although various methods exist to assess attention, the issue of noise and artifacts in EEG signals limits the practical application of these techniques, especially in portable single-channel devices (Grosselin et al., 2019). This study proposes an enhanced Extreme Learning Machine (ELM) algorithm that integrates L1/L2 norm regularization with DWT and employs ICA for preprocessing. Additionally, convex optimization techniques are utilized to enhance the Kalman filter, thereby improving signal estimation and model generalization. By combining advanced signal processing and machine learning methods, this research aims to enhance the accuracy and robustness of attention level estimation.

Experimental results indicate that the proposed model not only improves signal integrity but also achieves real-time adaptability, making it highly suitable for practical Brain-Computer Interface (BCI) applications. Compared to previous studies, the proposed algorithm demonstrates substantial superiority in real-time processing and noise reduction. Traditional EEG artifact (Jiang et al., 2019) removal methods typically require multi-channel EEG signals or synchronous acquisition of Electrooculography (EOG) signals as references and are predominantly offline batch processing methods, leading to delays and a lack of real-time application capabilities. Adaptive filtering methods and the Kalman filter face challenges in handling real-time, nonlinear, and non-smooth signals. The Kalman filter tends to treat sparse noise as Gaussian noise (Roth et al., 2017), whereas adaptive filtering methods have the problem of overfiltering in the filtering wave (Wang et al., 2023). In contrast, the robust Kalman algorithm introduced in this study can effectively mitigate noise interference through real-time adjustment of filter parameters while preserving the integrity of the original signal. These findings have profound implications for the application of BCI technology in fields such as education and healthcare, providing valuable insights for both theoretical advancement and practical application (Bonci et al., 2021). For example, the adaptive characteristics of the robust Kalman filter make it particularly suitable for real-time applications, ensuring minimal delay and high responsiveness, while effectively handling sparse noise to enhance signal integrity and retain essential signal components necessary for accurate attention assessment. Furthermore, the observed robust generalization capabilities across various datasets underscore its broad potential in a range of BCI applications, from educational tools to medical diagnostics.

The study acknowledges certain limitations, particularly the reliance on a relatively small participant group, which may affect the generalizability of the results. The small sample size, which does not encompass individuals from different age groups and health statuses, may limit the applicability of the results to a broader population (Vasileiou et al., 2018). Additionally, despite the high accuracy achieved by the proposed algorithm in signal processing, the inherent variability of EEG signals in real-world environments may significantly impact its performance (Souza and Naves, 2021). Future research should aim to validate the robustness and applicability of the algorithm using larger and more diverse datasets. Furthermore, future research can explore the findings from multiple perspectives, such as increasing the sample size and incorporating more diverse populations to enhance the robustness of the results. Additionally, applying the robust Kalman filter in other domains, such as mental health monitoring, cognitive training, and neurofeedback, could provide a deeper understanding of the method’s capabilities. Through these additional studies, we hope to enrich the understanding of this field and offer practical recommendations.

## Conclusion

The robust Kalman processing algorithm proposed in this paper demonstrates excellent performance in tracking and assessing attention levels. This algorithm utilizes convex optimization techniques on top of the Kalman filter to eliminate noise and obtain optimal estimates, followed by secondary corrections using extreme learning machines based on L1/L2 norms to improve system robustness and generalization. Experimental results show that the model achieves a maximum Test_AUC of 0.8167 and an average Test_AUC of 0.8678 on self-collected single-channel datasets, as well as a maximum Test_AUC of 0.8950 and an average Test_AUC of 0.8344 on the AF3 channel of public datasets. These results outperform traditional comparative algorithms, laying the foundation for EEG feature extraction and classification research.

The success of attention classification using single-channel EEG in this study highlights the importance of signal quality. Single-channel EEG provides a more efficient and portable solution without compromising accuracy. This makes it especially useful for real-time and practical applications in education, healthcare, and other fields.

## Data Availability

Publicly available datasets were analyzed in this study. This data can be found at: https://www.kaggle.com/datasets/inancigdem/eeg-data-for-mental-attention-state-detection/data.
